# Hourly differences in air pollution and risk of respiratory disease in the elderly: a time-stratified case-crossover study

**DOI:** 10.1186/1476-069X-13-67

**Published:** 2014-08-13

**Authors:** Takashi Yorifuji, Etsuji Suzuki, Saori Kashima

**Affiliations:** 1 Department of Human Ecology, Graduate School of Environmental and Life Science, Okayama University, 3-1-1 Tsushima-naka, Kita-ku, Okayama 700-8530, Japan; 2 Department of Epidemiology, Graduate School of Medicine, Dentistry and Pharmaceutical Sciences, Okayama University, 2-5-1 Shikata-cho, Kita-ku, Okayama 700-8558, Japan; 3 Department of Public Health and Health Policy, Institute of Biomedical & Health Sciences, Hiroshima University, 1-2-3 Kasumi, Minami-ku, Hiroshima 734-8551, Japan

**Keywords:** Air pollution, Ozone, Particulate matter, Respiratory disease, Short-term effect, Sulfur dioxide

## Abstract

**Background:**

Epidemiological studies have shown adverse effects of short-term exposure to air pollution on respiratory disease outcomes; however, few studies examined this association on an hourly time scale. We evaluated the associations between hourly changes in air pollution and the risk of respiratory disease in the elderly, using the time of the emergency call as the disease onset for each case.

**Methods:**

We used a time-stratified case-crossover design. Study participants were 6,925 residents of the city of Okayama, Japan, aged 65 or above who were taken to hospital emergency rooms between January 2006 and December 2010 for onset of respiratory disease. We calculated city-representative hourly average concentrations of air pollutants from several monitoring stations. By using conditional logistic regression models, we estimated odds ratios per interquartile-range increase in each pollutant by exposure period prior to emergency call, adjusting for hourly ambient temperature, hourly relative humidity, and weekly numbers of reported influenza cases aged ≥60.

**Results:**

Suspended particulate matter (SPM) exposure 24 to <72 hours prior to the onset and ozone exposure 48 to <96 hours prior to the onset were associated with the increased risk of respiratory disease. For example, following one interquartile-range increase, odds ratios were 1.05 (95% confidence interval: 1.01, 1.09) for SPM exposure 24 to <48 hours prior to the onset and 1.13 (95% confidence interval: 1.04, 1.23) for ozone exposure 72 to <96 hours prior to the onset. Sulfur dioxide (SO_2_) exposure 0 to <24 hours prior to onset was associated with the increased risk of pneumonia and influenza: odds ratio was 1.07 per one interquartile-range increase (95% confidence interval: 1.00, 1.14). Elevated risk for pneumonia and influenza of SO_2_ was observed at shorter lags (i.e., 8–18 hours) than the elevated risks for respiratory disease of SPM or ozone. Overall, the effect estimates for chronic obstructive pulmonary disease and allied conditions were equivocal.

**Conclusions:**

This study provides further evidence that hourly changes in air pollution exposure increase the risks of respiratory disease, and that SO_2_ may be related with more immediate onset of the disease than other pollutants.

## Background

A large number of epidemiological studies have shown adverse effects of short-term exposure to air pollution on respiratory disease outcomes [1-5], although most epidemiological studies to date have evaluated the association between daily air pollution and daily counts of respiratory outcomes. For example, a recent multi-city study across Europe and North America examined the effect of daily exposure levels (e.g., lag 1 day, average of lags 0 and 1 days) on daily mortality or hospitalizations [6]. Several studies therefore attempted to examine the effect of alternative pollutant metrics other than the 24-hour average on respiratory outcomes. Darrow et al. evaluated the different temporal metrics of air pollution (e.g., a daily 1-hour maximum, a day-time average, a night-time average) on the following days’ respiratory emergency visits and found that the different temporal metrics were also associated with the risk of visits [7]. Moreover, Stieb et al. examined the association between 3-hour averages of emergency visits and 3-hour average pollutant concentrations lagged up to 12 hours before, but found no association [8]. These attempts are, however, rare; thus, the epidemiological evidence on the association at an hourly temporal resolution between exposure to air pollution and the risk of respiratory outcomes is limited.

A recent study suggested that air pollution exposure caused more immediate effects on cardiovascular diseases and more delayed effects on respiratory diseases [9]. Indeed, a small number of epidemiological studies have examined the effect of hourly variations in air pollution on cardiovascular outcomes (such as ischemic heart disease [10,11], ischemic stroke [12-14], or hemorrhagic stroke [15] and demonstrated possible immediate adverse effects (e.g., 1–6 hours later [10]). These transient increases in risk immediately following air pollution exposure are supported by experimental studies [16,17]. Similarly, several experimental studies have demonstrated possible pulmonary function decrements immediately after air pollution exposure and pulmonary inflammation responses 16 to 18 hours after the exposure [18,19]. However, epidemiological studies so far cannot provide further evidence on these finer temporal associations (i.e., hourly time-scale) between air pollution and respiratory outcomes.

We therefore evaluated the associations between hourly changes in air pollution and the risk of respiratory disease onset in residents of Okayama, Japan, who had visited emergency rooms between January 2006 and December 2010. The findings may provide additional insights into the physiological mechanisms of air pollution health effects.

## Methods

### Study subjects

The Ambulance Division of the Fire Bureau in the city of Okayama provided electronic data (stripped of patient names) on all ambulance calls during the study period [20]. The call was made by the patients themselves or by others to request an ambulance. From the data, we selected 110,110 residents who had been brought to emergency rooms by ambulance between January 2006 and December 2010 in Okayama, a city located in the western part of Japan with population of 709,584 (151,140 people aged 65 or above) and an area of 790 km^2^ (in 2010). We restricted study participants to 6,925 patients aged 65 or above who were brought to an emergency room because of respiratory disease. We focused on the elderly because they are considered to be at greater risk of the adverse health effects of outdoor air pollution [2]. Because we could not differentiate each patient on the data, we might have counted the same participants more than once. The type of disease was diagnosed by physicians at the emergency rooms of the hospitals to which the patients were transported. Because we did not have the exact times of the disease onsets, we used the time of the emergency call as the disease onset for each case.

### Air pollution, meteorological, and influenza data

We obtained hourly concentrations of suspended particulate matter (SPM), nitrogen dioxide (NO_2_), sulfur dioxide (SO_2_), ozone, and carbon monoxide (CO), measured at monitoring stations in the city of Okayama during the study period, from the Okayama Prefectural Government. Particulate matter (PM) is measured as SPM in Japan and accounts for PM with an aerodynamic diameter less than 7 μm (PM_7_). During the study period, 11 stations were used for SPM measurements, 11 for NO_2_, seven for SO_2_, eight for ozone, and two for CO. The entire area of the city is covered by 30-km buffers from each monitoring station. We then calculated city-representative hourly average concentrations of each air pollutant from hourly concentrations at each monitoring station. When hourly concentrations at several monitoring stations were not available, we used hourly concentrations at other stations where the data was available to calculate the city-representative hourly average concentrations. Despite these imputations, we lacked 502 hourly concentrations for ozone (1.15% of eligible hours) and 26 for CO (0.06% of eligible hours) during the study period.

We also obtained hourly temperature and relative humidity during the study period from one weather station in the city of Okayama managed by the Japan Metrological Agency. There were no missing data for temperature and relative humidity.

We obtained weekly numbers of reported influenza cases among monitoring medical institutions in the city from the website of the public health center in the Okayama city [21].

### Health outcomes

The type of disease was diagnosed by medical doctors at the emergency rooms of the hospitals to which the patients were transported, and coded in accordance with the 10^th^ International Classification of Disease (ICD-10). We used the following diseases as main outcomes based on the previous studies [22]: respiratory disease (ICD-10: J00-99), pneumonia and influenza (J10-22), and chronic obstructive pulmonary disease (COPD) and allied conditions (J40-47).

### Statistical analyses

We used a time-stratified, case-crossover design. A case-crossover design can be considered as a case–control version of a cross-over study and can adjust for time-invariant confounders [23]. The design uses cases only; for each individual case, exposure before the event (case period) is compared with exposure at other control (or “referent”) periods. Time-stratified referent selection is recommended to ensure unbiased estimates from conditional logistic regression and to avoid bias resulting from time trend [23]; we thus selected control periods from the same times on other days, on the same days of the week in the same months and years (e.g., the same six-hour intervals on other days, on the same days of the week in the same months and years as those on which the case occurred).

We conducted conditional logistic regression analyses to estimate adjusted odds ratios (ORs) and 95% confidence intervals (CIs) for the association between air pollution exposure and each health outcome. We used exposure data as continuous variables and estimated adjusted ORs for an interquartile range (IQR) increase in each air pollutant during the study periods. In all analyses, we adjusted for hourly ambient temperature using a natural spline with 6 degrees of freedom (df), hourly relative humidity with 3 df, and weekly numbers of reported influenza cases aged ≥60 among monitoring medical institutions in the city. We chose the number of df following a previous air pollution study in Japan [22] and because the relationship between temperature or humidity and morbidity is considered non-linear. We used meteorological data at the time of the case event.

We first evaluated the effect of exposure to each pollutant, averaged during eight different periods prior to the case event (i.e., emergency calls) (0 to <6 hours, 6 to <12 hours, 12 to <18 hours, 18 to <24 hours, 0 to <24 hours, 24 to <48 hours, 48 to <72 hours, and 72 to <96 hours), on health outcomes (respiratory disease, pneumonia and influenza, and COPD and allied conditions). In addition, we applied quadratic distributed lag models to estimate the cumulative effects of the current and the 3 previous days (i.e., <96 hours before) (lags 0–3 days) instead of applying single lag models [24]. We further performed a multi-pollutant model to differentiate the role of each pollutant if some of the pollutants were associated with the same outcomes.

To evaluate the effect of shorter exposure in detail, we evaluated the effect of selected air pollutants (which were associated with health outcomes in the single-pollutant models) averaged for 2-hour increments prior to the case event (i.e., 0 to <2 hours through 46 to <48 hours). We included all periods in the model individually.

In additional analyses, we evaluated whether the effects of these selected pollutants were modified by patient characteristics: age (<75 years vs. ≥75 years), sex, time of onset (8 am to 7 pm vs. 8 pm to 7 am), history of hypertension, history of coronary heart disease, history of cerebrovascular disease, history of diabetes mellitus, and history of respiratory disease. The information on comorbidity was obtained from the patients themselves or their relatives by ambulance personnel. The p values for interaction less than 0.05 were considered significant.

In sensitivity analyses, to reduce exposure measurement error, we excluded two areas located in the northern part of the city of Okayama (the towns of Takebe and Mitsu) so that the study area could be covered by 20-km buffers from each monitoring station. In addition, we repeated the analyses without adjusting for weekly numbers of reported influenza cases. We also excluded influenza cases from the health outcome of pneumonia and influenza and repeated the analysis. Finally, we changed number of dfs for hourly ambient temperature and hourly relative humidity to examine the robustness of the results.

We conducted analyses using the R statistical package version 2.15.0 [25]. The Institutional Review Board of Graduate School of Medicine, Dentistry and Pharmaceutical Sciences, Okayama University approved this study on 26 June 2012 (No. 556).

## Results

Table 1 presents the characteristics of the participants. More than 20% of the patients had a history of respiratory disease, and about half of the emergency room visits were for pneumonia and influenza.

**Table 1:** Characteristics of emergency hospital visits of people over 65 years of age with respiratory disease residing in Okayama-city, Japan, 2006–2010 (n = 6,925)

	**Patients**
Mean Age (year) (SD)	82 (8.3)
Sex (% women)^a^	3003 (43.4)
Emergency call (% daytime, 8 am to 8 pm)^a^	4871 (70.3)
Medical history^a^	
Hypertension	559 (8.1)
Coronary heart disease	209 (3.0)
Cerebrovascular disease	1012 (14.6)
Diabetes mellitus	369 (5.3)
Respiratory disease	1574 (22.7)
Other diseases	2292 (33.1)
None	459 (6.6)
Unknown	451 (6.5)
Types of respiratory disease^a^	
Pneumonia and influenza	3227 (46.6)
COPD and allied conditions	767 (11.1)
Acute upper respiratory infections	297 (4.3)
Other diseases of upper respiratory tract	303 (4.4)
Lung diseases due to external agents	1118 (16.1)
Other respiratory diseases principally affecting the interstitium	120 (1.7)
Suppurative and necrotic conditions of lower respiratory tract	15 (0.2)
Other diseases of pleura	264 (3.8)
Other diseases of the respiratory system	814 (11.8)

^a^No.(%) of participants is shown.

*COPD*; Chronic obstructive pulmonary disease, *SD*; Standard deviation.

During the study period, the IQRs of air pollutants were 20.6 μg/m^3^ for SPM, 11.1 ppb for NO_2_, 2.3 ppb for SO_2_, 25.8 ppb for ozone, and 0.3 ppm for CO (Table 2). Concentrations for SPM, SO_2_, and ozone were higher in the daytime, while concentration for NO_2_ was higher in the nighttime. Hourly SPM was moderately correlated with other pollutants except ozone. By contrast, ozone was weakly correlated with SO_2_ or inversely associated with NO_2_ or CO. Averages of temperature and relative humidity were 16.7°C and 65.3%, respectively.

**Table 2:** Characteristics and Spearman correlation coefficients for hourly air pollutants and meteorological variables

	**Mean (SD)**			**IQR**	**SPM**	**NO**_**2**_	**SO**_**2**_	**Ozone**	**CO**	**Temperature**	**Relative humidity**
	**All day**	**Daytime (8 am to 7 pm)**	**Nighttime (8 pm to 7 am)**								
SPM (μg/m^3^)	26.8 (18.3)	27.5 (18.4)	26.1 (18.1)	20.6	1	0.41	0.50	0.06	0.35	0.23	0.00
NO_2_ (ppb)	17.1 (8.1)	15.9 (7.9)	18.3 (8.2)	11.1		1	0.40	−0.40	0.63	−0.26	0.20
SO_2_ (ppb)	3.0 (2.5)	3.7 (3.0)	2.3 (1.7)	2.3			1	0.26	0.19	0.22	−0.31
Ozone (ppb)	25.9 (17.9)	33.8 (18.5)	18.1 (13.1)	25.8				1	−0.31	0.36	−0.65
CO (ppm)	0.6 (0.3)	0.6 (0.3)	0.6 (0.3)	0.3					1	−0.17	0.17

*CO*, Carbon monoxide; *IQR*, Interquartile range; *NO*_*2*_, Nitrogen dioxide; *SD*, Standard deviation; *SO*_*2*_, Sulfate dioxide; *SPM*, Suspended particulate matter.

When we examined the effect of each pollutant averaged at eight different periods prior to the case event, SPM (24 to <72 hours prior to the onset) and ozone (48 to <96 hours prior to the onset) exposures were associated with the risk of respiratory disease (Table 3). Following one IQR increase, ORs were 1.05 (95% CI: 1.01, 1.09) for SPM exposure 24 to <48 hours prior to the onset and 1.13 (95% CI: 1.04, 1.23) for ozone exposure 72 to <96 hours prior to the onset. The point estimates from distributed lag models were also elevated for both pollutants. For pneumonia and influenza, the highest and the most precise OR was observed for SO_2_ exposure 0 to <24 hours prior to onset (OR = 1.07, 95% CI: 1.00, 1.14). In contrast, the effect estimates for COPD and allied conditions were equivocal. Even in the two-pollutant model, which adjusted for SPM and ozone at the same exposure periods prior to the onset, the results for respiratory disease did not change (data not shown). Moreover, even if we adjusted for both SPM and ozone exposures 0 to <24 hours prior to onset, the OR for pneumonia and influenza was still elevated for SO_2_ exposure 0 to <24 hours prior to onset (OR = 1.07, 95% CI: 0.99, 1.17).

**Table 3:** **Adjusted OR and 95% CI per interquartile-range increase**^**a**^**in each pollutant by exposure period prior to emergency call**

	**Respiratory disease**	**Pneumonia and influenza**	**COPD and allied conditions**
**SPM**			
0 to <6 hours	1.03 (0.99, 1.06)	1.02 (0.97, 1.07)	1.06 (0.96, 1.17)
6 to <12 hours	1.03 (0.99, 1.06)	1.03 (0.98, 1.09)	1.02 (0.93, 1.12)
12 to <18 hours	1.02 (0.98, 1.05)	1.01 (0.96, 1.06)	1.00 (0.90, 1.11)
18 to <24 hours	1.02 (0.98, 1.05)	1.01 (0.96, 1.07)	0.97 (0.87, 1.07)
0 to <24 hours	1.03 (0.99, 1.07)	1.03 (0.97, 1.09)	1.02 (0.91, 1.14)
24 to <48 hours	1.05 (1.01, 1.09)	1.05 (0.99, 1.11)	0.99 (0.88, 1.10)
48 to <72 hours	1.03 (1.00, 1.07)	1.01 (0.96, 1.07)	0.98 (0.88, 1.09)
72 to <96 hours	1.01 (0.97, 1.05)	0.97 (0.91, 1.02)	0.90 (0.80, 1.02)
Distributed lags^b^	1.05 (1.00, 1.12)	1.01 (0.93, 1.10)	0.93 (0.79, 1.10)
**NO**_**2**_			
0 to <6 hours	1.02 (0.97, 1.08)	1.05 (0.97, 1.14)	0.97 (0.83, 1.13)
6 to <12 hours	1.04 (0.98, 1.09)	1.06 (0.98, 1.15)	1.03 (0.88, 1.20)
12 to <18 hours	1.03 (0.98, 1.08)	1.03 (0.96, 1.11)	0.99 (0.85, 1.15)
18 to <24 hours	1.02 (0.97, 1.07)	1.02 (0.95, 1.09)	0.89 (0.77, 1.04)
0 to <24 hours	1.04 (0.98, 1.11)	1.06 (0.97, 1.17)	0.95 (0.78, 1.15)
24 to <48 hours	1.05 (0.99, 1.11)	1.06 (0.97, 1.16)	1.01 (0.84, 1.22)
48 to <72 hours	1.02 (0.96, 1.08)	1.02 (0.94, 1.11)	0.96 (0.80, 1.14)
72 to <96 hours	0.98 (0.92, 1.04)	0.93 (0.86, 1.02)	0.84 (0.70, 0.99)
Distributed lags^b^	1.05 (0.95, 1.15)	1.03 (0.90, 1.19)	0.83 (0.63, 1.10)
**SO**_**2**_			
0 to <6 hours	1.01 (0.98, 1.05)	1.02 (0.98, 1.07)	1.02 (0.92, 1.13)
6 to <12 hours	1.02 (0.98, 1.06)	1.05 (0.99, 1.11)	0.96 (0.86, 1.07)
12 to <18 hours	1.03 (1.00, 1.07)	1.06 (1.00, 1.12)	1.04 (0.94, 1.15)
18 to <24 hours	1.01 (0.98, 1.04)	1.03 (0.99, 1.07)	0.99 (0.91, 1.08)
0 to <24 hours	1.03 (0.99, 1.08)	1.07 (1.00, 1.14)	1.00 (0.88, 1.14)
24 to <48 hours	1.03 (0.98, 1.07)	1.05 (0.98, 1.11)	0.97 (0.86, 1.10)
48 to <72 hours	1.02 (0.98, 1.07)	1.02 (0.96, 1.08)	0.94 (0.83, 1.06)
72 to <96 hours	1.01 (0.96, 1.05)	0.96 (0.90, 1.03)	0.85 (0.74, 0.96)
Distributed lags^b^	1.05 (0.98, 1.11)	1.04 (0.95, 1.15)	0.85 (0.71, 1.03)
**Ozone**			
0 to <6 hours	1.01 (0.94, 1.09)	0.94 (0.84, 1.05)	1.10 (0.88, 1.39)
6 to <12 hours	1.02 (0.94, 1.09)	0.96 (0.86, 1.07)	1.04 (0.84, 1.29)
12 to <18 hours	1.04 (0.97, 1.12)	1.00 (0.90, 1.11)	1.15 (0.93, 1.43)
18 to <24 hours	1.02 (0.96, 1.09)	0.99 (0.90, 1.09)	1.27 (1.03, 1.55)
0 to <24 hours	1.04 (0.95, 1.15)	0.95 (0.83, 1.10)	1.29 (0.96, 1.74)
24 to <48 hours	0.98 (0.89, 1.06)	0.87 (0.76, 0.99)	1.00 (0.76, 1.32)
48 to <72 hours	1.09 (1.00, 1.19)	1.00 (0.88, 1.13)	0.98 (0.75, 1.28)
72 to <96 hours	1.13 (1.04, 1.23)	1.06 (0.93, 1.20)	1.21 (0.93, 1.58)
Distributed lags^b^	1.15 (1.01, 1.32)	0.94 (0.77, 1.14)	1.36 (0.89, 2.09)
**CO**			
0 to <6 hours	1.00 (0.96, 1.05)	1.01 (0.94, 1.07)	0.99 (0.87, 1.12)
6 to <12 hours	1.00 (0.95, 1.04)	1.01 (0.94, 1.08)	0.93 (0.82, 1.06)
12 to <18 hours	0.99 (0.95, 1.04)	0.99 (0.93, 1.05)	0.96 (0.84, 1.09)
18 to <24 hours	0.99 (0.95, 1.04)	0.99 (0.93, 1.05)	0.89 (0.78, 1.01)
0 to <24 hours	0.99 (0.94, 1.05)	0.99 (0.91, 1.07)	0.90 (0.76, 1.07)
24 to <48 hours	1.03 (0.98, 1.09)	1.05 (0.97, 1.13)	0.96 (0.82, 1.12)
48 to <72 hours	1.00 (0.95, 1.05)	0.99 (0.92, 1.07)	0.97 (0.84, 1.12)
72 to <96 hours	0.94 (0.90, 0.99)	0.94 (0.88, 1.01)	0.81 (0.70, 0.94)
Distributed lags^b^	0.96 (0.89, 1.04)	0.95 (0.85, 1.07)	0.78 (0.61, 0.99)

^a^Adjusted for ambient temperature (df = 6), relative humidity (df = 3), and weekly numbers of reported influenza cases aged ≥60. Interquartile ranges are 20.6 μg/m^3^ for SPM, 11.1 ppb for NO_2_, 2.3 ppb for SO_2_, 25.8 ppb for ozone, and 0.3 ppm for CO.

^b^Quadratic distributed lag (0 to lag 3).

*CI*, Confidence interval; *CO*, Carbon monoxide; *COPD*; Chronic obstructive pulmonary disease, *NO*_*2*_, Nitrogen dioxide; *OR*, Odds ratio; *SO*_*2*_, Sulfate dioxide; *SPM*, Suspended particulate matter.

**Figure 1:**
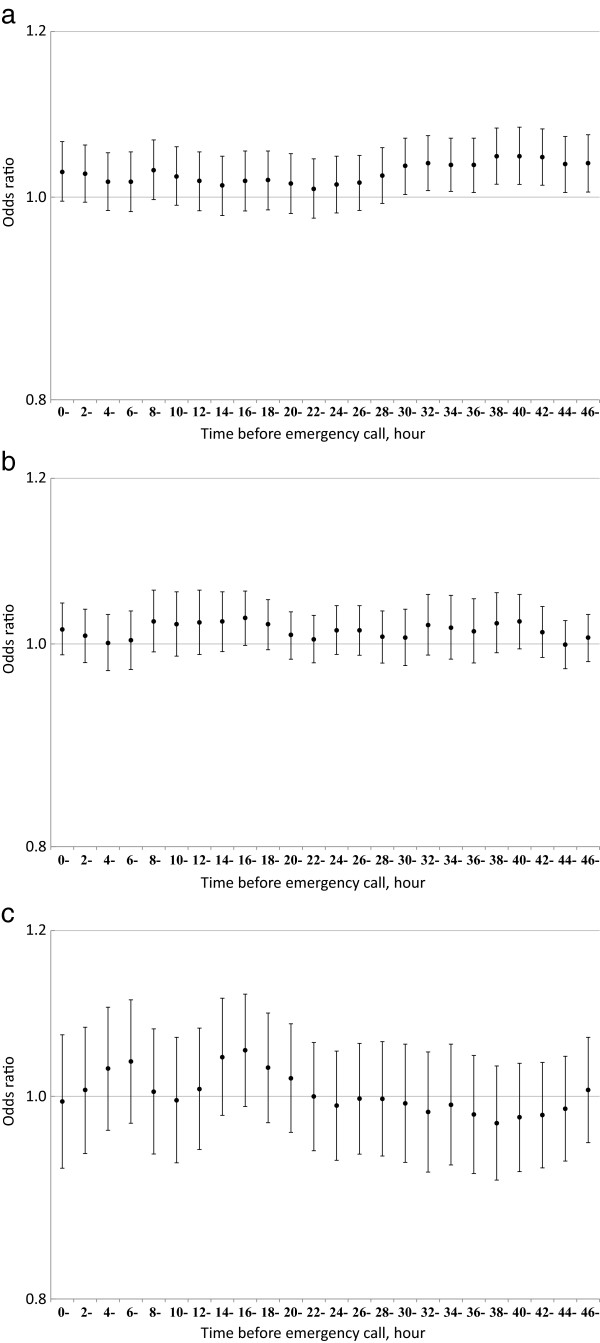
**Odds ratios of respiratory disease for an interquartile range increase in (a) suspended particulate matter (20.6 μg/m**^**3**^**), (b) sulfur dioxide (2.3 ppb), and (c) ozone (25.8 ppb) in the hours prior to disease onset.** Vertical bars indicate 95% confidence intervals.

**Figure 2:**
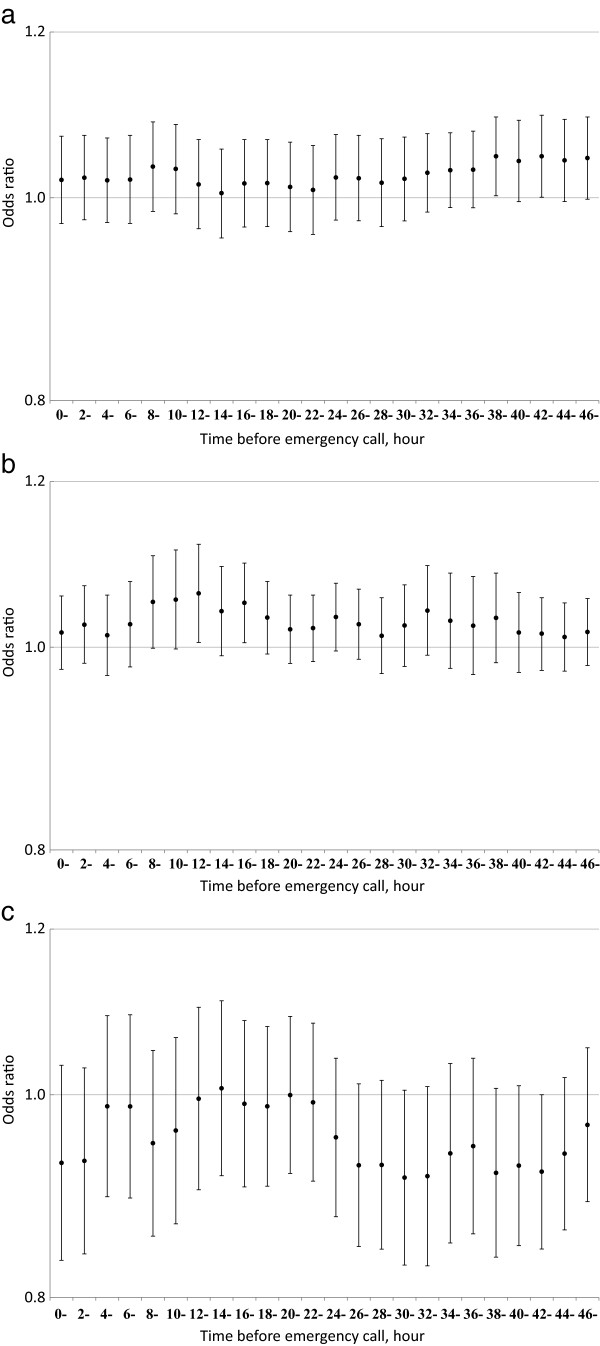
**Odds ratios of pneumonia and influenza for an interquartile range increase in (a) suspended particulate matter (20.6 μg/m**^**3**^**), (b) sulfur dioxide (2.3 ppb), and (c) ozone (25.8 ppb) in the hours prior to disease onset.** Vertical bars indicate 95% confidence intervals.

We examined the effect of exposure within 48 hours of the onset of respiratory disease in more detail (Figure 1): SPM exposure 30–48 hours before onset was associated with elevated risks of respiratory disease, while ozone exposure was not associated with the elevated risk of respiratory disease within 48 hours of the onset as expected from Table 3. By contrast, SO_2_ exposure was associated with the elevated risk of pneumonia and influenza in smaller number of hours prior to the onset (e.g., 8–18 hours) (Figure 2).

In stratified analyses by patient characteristics (Table 4), effect estimates for the association between ozone exposure and respiratory disease were higher for women and those without a history of diabetes mellitus. In addition, effect estimates for the association of SO_2_ exposure with pneumonia and influenza were higher for those with a history of hypertension and those without histories of cerebrovascular and respiratory diseases. However, a statistically significant interaction was observed only for the stratification by a disease history of respiratory disease for the association of SO_2_ with pneumonia and influenza.

**Table 4:** **Associations of an interquartile-range increase in SPM (24–48 hours), ozone (72–96 hours), and SO**_**2 **_**(0–24 hours) prior to emergency call with health outcomes in each subgroup of patients**

	**Respiratory disease**				**Pneumonia and influenza**	
	**SPM (24–48 hours)**		**Ozone (72–96 hours)**		**SO**_**2 **_**(0–24 hours)**	
	**OR (95% CI)**^**a**^	***p***^**b**^	**OR (95% CI)**^**a**^	***p***^**b**^	**OR (95% CI)**^**a**^	***p***^**b**^
**Age (year)**						
≥75	1.06 (1.02,1.10)	0.33	1.13 (1.03, 1.24)	0.93	1.08 (1.01, 1.16)	0.25
<75	1.01 (0.93,1.10)		1.14 (0.94,1.38)		0.99 (0.85,1.14)	
**Sex**						
Men	1.07 (1.02,1.12)	0.25	1.07 (0.95,1.19)	0.12	1.04 (0.95,1.13)	0.30
Women	1.02 (0.97,1.08)		1.22 (1.07,1.39)		1.10 (1.01,1.21)	
**Onset**						
8 am to 7 pm	1.05 (1.00,1.09)	0.69	1.12 (1.01,1.24)	0.79	1.07 (0.99,1.15)	1.00
8 pm to 7 am	1.06 (0.99,1.14)		1.15 (0.98,1.35)		1.07 (0.94,1.21)	
**Past history**						
Hypertension						
Yes	1.08 (0.95,1.23)	0.63	1.28 (0.95,1.74)	0.37	1.25 (1.00,1.56)	0.13
No	1.05 (1.01,1.09)		1.11 (1.01,1.22)		1.05 (0.98,1.12)	
Coronary heart disease						
Yes	1.17 (0.94,1.46)	0.35	1.37 (0.85,2.23)	0.41	1.09 (0.77,1.53)	0.90
No	1.05 (1.01,1.09)		1.12 (1.02,1.22)		1.06 (1.00,1.14)	
Cerebrovascular disease						
Yes	1.03 (0.93,1.14)	0.61	1.01 (0.81,1.25)	0.28	0.94 (0.81,1.09)	0.08
No	1.06 (1.01,1.10)		1.15 (1.04,1.26)		1.09 (1.02,1.17)	
Diabetes mellitus						
Yes	0.99 (0.84,1.18)	0.50	0.85 (0.58,1.24)	0.14	0.98 (0.78,1.23)	0.46
No	1.05 (1.02,1.10)		1.14 (1.04,1.25)		1.07 (1.00,1.14)	
Respiratory disease						
Yes	1.01 (0.94,1.09)	0.24	1.15 (0.96,1.37)	0.80	0.92 (0.79,1.08)	0.04
No	1.07 (1.02,1.11)		1.12 (1.01,1.23)		1.09 (1.02,1.17)	

^a^Adjusted for ambient temperature (df = 6), relative humidity (df = 3), and weekly numbers of reported influenza cases aged ≥60. Interquartile ranges are 20.6 μg/m^3^ for SPM, 2.3 ppb for SO_2_, and 25.8 ppb for ozone.

^b^*p* value for interaction.

*CI*, Confidence interval; *SO*_*2*_, Sulfate dioxide; *SPM*, Suspended particulate matter.

In sensitivity analyses, exclusion of the towns of Takebe and Mitsu from the analyses provided similar results: e.g., ORs for respiratory disease were 1.05 (95% CI: 1.01, 1.09) for SPM exposure 24 to <48 hours prior to the case events and 1.13 (95% CI: 1.04, 1.23) for ozone exposure 72 to <96 hours prior to the case events and OR for pneumonia and influenza was 1.06 (95% CI: 1.00, 1.13) for SO_2_ exposure 0 to <24 hours prior to the case events. Not adjusting for weekly numbers of reported influenza cases also provided similar results (data not shown), which implies that influenza epidemics did not confound the association between short-term exposure to air pollution and respiratory health effects. Moreover, when we excluded influenza cases (n = 111) from the analysis, OR for pneumonia and influenza was 1.06 (95% CI: 1.00, 1.14) for SO_2_ exposure 0 to <24 hours prior to the case events. Finally, when we adopted different numbers of dfs (hourly ambient temperature with 8 dfs and hourly relative humidity with 5 dfs), the results did not change (data not shown).

## Discussion

In the present study, we evaluated the associations between hourly changes in air pollution and the risk of respiratory disease onset in Okayama, Japan, using the time of the emergency call as the disease onset for each case. We found that SPM (PM_7_) exposure 24 to <72 hours prior to the onset and ozone exposure 48 to <96 hours prior to the onset were associated with the increased risk of respiratory disease, while SO_2_ exposure 0 to <24 hours prior to onset was associated with the increased risk of pneumonia and influenza. Indeed, elevated risk for pneumonia and influenza of SO_2_ was observed at shorter lags (i.e., 8–18 hours) than the elevated risks for respiratory disease of SPM or ozone.

The observed respiratory effect of SPM, ozone, and SO_2_ are consistent with previous findings [2,5]. To interpret the finding, one point should be noted that SPM (PM_7_) are particles larger than PM with less than 2.5 μm (PM_2.5_) but smaller than PM with less than 10 μm (PM_10_); thus, SPM is also included in the range of respirable fraction. A large number of studies have shown that PM (both PM_2.5_ and PM_10_) and ozone are associated with an increased risk of respiratory mortality or morbidity [1,5,26]. The consistency of the effects of SO_2_ is less than that of PM or ozone. However, meta-analytic studies or multi-city studies have consistently shown that SO_2_ also has adverse effects on respiratory outcomes [5,27-29], which are supported by intervention studies in Hong Kong, where an immediate fall in ambient SO_2_ was observed following restricting sulfur in fuel [30,31].

Different lag structures (i.e., 8–18 hours for SO_2_; 1–2 days for SPM; and 2–3 days for ozone) for respiratory outcomes merit consideration. The lags for SPM or ozone are plausible considering the previous studies [6,8,22,32]. The shorter lag for SO_2_ may be supported by an experimental study which demonstrated a mild increase in neutrophils in the fluid obtained by bronchoalveolar lavage 18 hours after concentrated ambient air particles exposure [18,33]. Basically, SO_2_ can be converted to sulfuric acid and contribute to the formation of PM [34]. In the experimental study we mentioned, sulfuric acid was highly correlated with the particles. Although it may be difficult to attribute this immediate increased risk SO_2_ itself, PM may become more toxic and have immediate effects when SO_2_ coexists and gets absorbed onto PM surfaces [5].

Compared to respiratory disease or pneumonia and influenza, the findings for COPD and allied conditions were equivocal, which is inconsistent with the previous studies [2]. This is probably due to small number of cases in the present study. Because we could not include patients who arrived at hospitals by their own means, this may reduce the number of the COPD cases in the present study.

We also observed that several patient characteristics were associated with increased risk of air pollution. The higher effect estimate for women of the association between ozone exposure and respiratory disease is consistent with a multi-city study in Asia [35], but a study in Massachusetts, US, did not find effect modification by sex [36]. Among preexisting diseases, we observed a statistically significant interaction only for respiratory disease: those without a history of respiratory disease had a higher effect estimate for the association of SO_2_ exposure with pneumonia and influenza. Although the reason is unclear and we have no other evidence, those without respiratory disease may be more sensitive to the exposure or be exposed more heavily, probably because they can stay outdoors longer. However, a previous study with a cohort of COPD patients in Italy suggested those with preexisting disease history (in particular, heart conduction disorders and cerebrovascular disease) were more susceptible to air pollution [37]. Further investigation may be needed.

The strength of the present study is that we could obtain hourly data on both air pollution and emergency visits, which enabled us to examine the effect of hourly changes in air pollution on the risk of respiratory disease. Previous mortality studies were not able to determine whether air pollution could trigger new respiratory disease events or precipitate the deaths of patients with preexisting disease [4,5], and their use of a daily time scale prevented determination of an exact temporal relationship between air pollution exposure and disease onset. In addition, we were able to obtain individual information (e.g., preexisting disease), and thus could evaluate effect modifications of these individual characteristics.

However, several limitations should be noted. First, most of the reported associations were not statistically significant. Because of the large number of tests we conducted, there is a possibility of chance findings.

Second, because we did not have the exact times of disease onsets, we used the time of emergency call as the disease onset for each case. Compared with cardiovascular disease, the disease onset of respiratory disease would be difficult to identify and the disease progression is probably gradual. Thus, the disease onset may only indicate the appearance of respiratory symptoms rather than incidence of disease. The present finding that shorter lag for SO_2_ than for SPM or ozone may just show the differences in latent periods [38] between disease onset and the need for emergency call, i.e., the different speed in disease progression of the disease which these air pollutants may induce.

Third, disease diagnosis was made by physicians at emergency rooms of the hospitals to which the patients were transported. In general, emergency patients are brought to large hospitals in the city of Okayama, where diagnostic technique is standardized. Although we used a definition of pneumonia and influenza together, 97% of the patients (n = 3,116) were diagnosed pneumonia or lower respiratory infection (LRI); thus, the effect estimates obtained for pneumonia and influenza would be attributable to LRI. Indeed, when we excluded influenza cases from the analysis, the result did not change substantially.

A further limitation is that we assumed all residents were exposed to the same concentration without considering spatial distribution. However, the analyses restricted to 20-km buffers from each monitoring station provided similar effect estimates.

Finally, we did not include patients who arrived at hospitals by their own means. Therefore, we might not be able to generalize the present findings to all emergency hospital visits.

## Conclusions

The present study provides further evidence that hourly changes in air pollution exposure increase the risks of respiratory disease. Elevated risk for SO_2_ was observed at shorter lags (i.e., 8–18 hours) than the elevated risks for SPM or ozone (i.e., lags of 1–4 days); thus, SO_2_ may be related with more immediate onset of the disease than SPM or ozone. These findings, derived from finer temporal resolution of air pollution, should provide additional insights into the physiological mechanisms of air pollution health effects as well as air pollution regulations.

## Abbreviations

CI: Confidence interval; CO: Carbon monoxide; COPD: Chronic obstructive pulmonary disease; df: Degree of freedom; ICD: International Classification of Disease; IQR: Interquartile range; LRI: Lower respiratory infection; NO_2_: Nitrogen dioxide; OR: Odds ratio; PM: Particulate matter; SD: Standard deviation; SO_2_: Sulfate dioxide; SPM: Suspended particulate matter.

## Competing interests

The authors declare that they have no competing interest.

## Authors’ contributions

Study concept and design: TY, ES, SK; Data collection: ES; Data handling: TY, SK; Analysis: TY; Interpretation of data: TY, ES, SK; Drafting of the manuscript: TY; Critical revision of the manuscript: ES, SK. All authors read and approved the final manuscript.
